# Precision in Prosthetics: Two Case Reports on Transformative Rehabilitation With Custom Ocular and Orbital Prostheses

**DOI:** 10.7759/cureus.72485

**Published:** 2024-10-27

**Authors:** Yashika Bali, Ayush Kumar, Ravpreet Singh, Riddhi Sharma, Tarunpreet Gill, Vaishali Kalra

**Affiliations:** 1 Department of Prosthodontics and Crown and Bridge, Subharti Dental College and Hospital, Swami Vivekanand Subharti University, Meerut, IND; 2 Department of Prosthodontics and Crown and Bridge, National Dental College and Hospital, Mohali, IND; 3 Department of Prosthodontics and Crown and Bridge, Sandhu Dental Care Centre, Amritsar, IND; 4 Department of Prosthodontics and Crown and Bridge, Bhojia Dental College and Hospital, Solan, IND

**Keywords:** low-grade myxofibrosarcoma, maxillofacial defect, maxillofacial prosthesis, maxillo-facial trauma, retinoblastoma

## Abstract

The eyes play a crucial role in vision and emotional expression, and their loss can profoundly affect appearance and psychological well-being. Eye loss may result from trauma, tumors, infections, malignancies, or congenital abnormalities. Surgical methods for removing an eye include enucleation, evisceration, and exenteration. When surgical reconstruction is insufficient, prosthetic rehabilitation becomes necessary, requiring collaboration among ophthalmologists, oral and maxillofacial surgeons, and prosthodontists. Ocular and orbital prostheses, which may be either prefabricated or custom-made, help maintain hygiene and monitor for tumor recurrence, significantly improving the patient’s quality of life.

We report two cases that describe a technique for fabricating a custom ocular and silicone-based orbital prosthesis, particularly useful for patients with defects caused by tumors or trauma. One case involved a five-year-old boy who sought treatment for an ocular prosthesis after enucleation of his left eye due to retinoblastoma. A custom prosthesis was crafted to accommodate the child’s facial growth, and follow-up visits indicated positive aesthetic and psychological outcomes. Another case involved a 54-year-old man who had undergone exenteration of his left eye eight years prior due to low-grade myxofibrosarcoma. Extensive preoperative planning and patient counseling were undertaken, and a custom magnet-retained silicone-based orbital prosthesis was designed to match his facial features. Multiple adjustments were made for comfort and functionality, and the patient reported significant improvements in social interactions and self-esteem.

These cases underscore the importance of personalized care and teamwork in prosthetic rehabilitation. For many years, artificial eyes have been replaced with either stock or custom prostheses, with custom options generally providing a more accurate and aesthetically pleasing result, especially in patients who have undergone enucleation or exenteration. While implant-retained prostheses can enhance outcomes, conventional ocular and orbital prostheses remain practical, cost-effective options that support psychosocial well-being, even though they do not restore vision. In pediatric cases, it is critical to regularly adjust the prosthesis to accommodate the child’s growth and prevent complications. Room temperature vulcanized (RTV) silicone is favored for its flexibility and compatibility, though it requires routine maintenance due to wear and discoloration. Samarium-cobalt magnets, commonly used for retention, offer strong stability but can corrode over time, necessitating encapsulation and regular inspections.

Despite some limitations, traditional prostheses are highly effective in ocular rehabilitation, and advances in computer-aided designing-computer-aided manufacturing (CAD-CAM) technology now offer enhanced solutions for addressing such defects. Rehabilitating defects with custom prostheses is a transformative process, significantly improving patients' lives. By restoring natural appearance and function, these prostheses not only enhance physical aesthetics but also boost emotional well-being and social confidence. The precise creation and fitting of these custom devices ensure a perfect match, leading to greater comfort and patient satisfaction.

## Introduction

The eyes are not only vital for vision but also serve as significant indicators of a person's emotions and overall health. Their prominence in facial recognition often makes their loss particularly impactful on both aesthetic and psychological levels. The loss or absence of an eye can occur due to various reasons such as trauma, tumors, infections, malignancies, or congenital abnormalities. Surgical methods for eye removal are categorized into three main types, as described by Peyman, Saunders, and Goldberg: enucleation, evisceration, and exenteration [1].

Enucleation entails the removal of the eye globe along with a portion of the optic nerve. In contrast, evisceration involves extracting the contents of the eye while preserving the sclera and extraocular muscles. Exenteration, the most radical approach, includes the removal of the eye along with its adnexa and portions of the bony orbit [2]. When surgical reconstruction fails to yield satisfactory outcomes, prosthetic rehabilitation becomes the preferred treatment. A multidisciplinary approach that includes ophthalmologists, oral and maxillofacial surgeons, and maxillofacial prosthodontists is vital for effective rehabilitation and follow-up care [3]. Ocular and orbital prostheses can be either prefabricated or custom-made. Prosthetic rehabilitation serves as a viable alternative to surgical reconstruction, as these prostheses effectively mimic the missing structures while facilitating hygiene maintenance around the defect and allowing for monitoring of any potential tumor recurrence [4]. It ensures comprehensive care, enhances patient recovery, and improves quality of life by addressing both the physical and psychological impacts of orbital loss [5].

These case reports discuss a method for creating an ocular and silicone-based orbital prosthesis, which is particularly beneficial for patients with defects resulting from tumor or midfacial trauma.

## Case presentation

Case 1

Ocular Prosthesis

**Figure 1:**
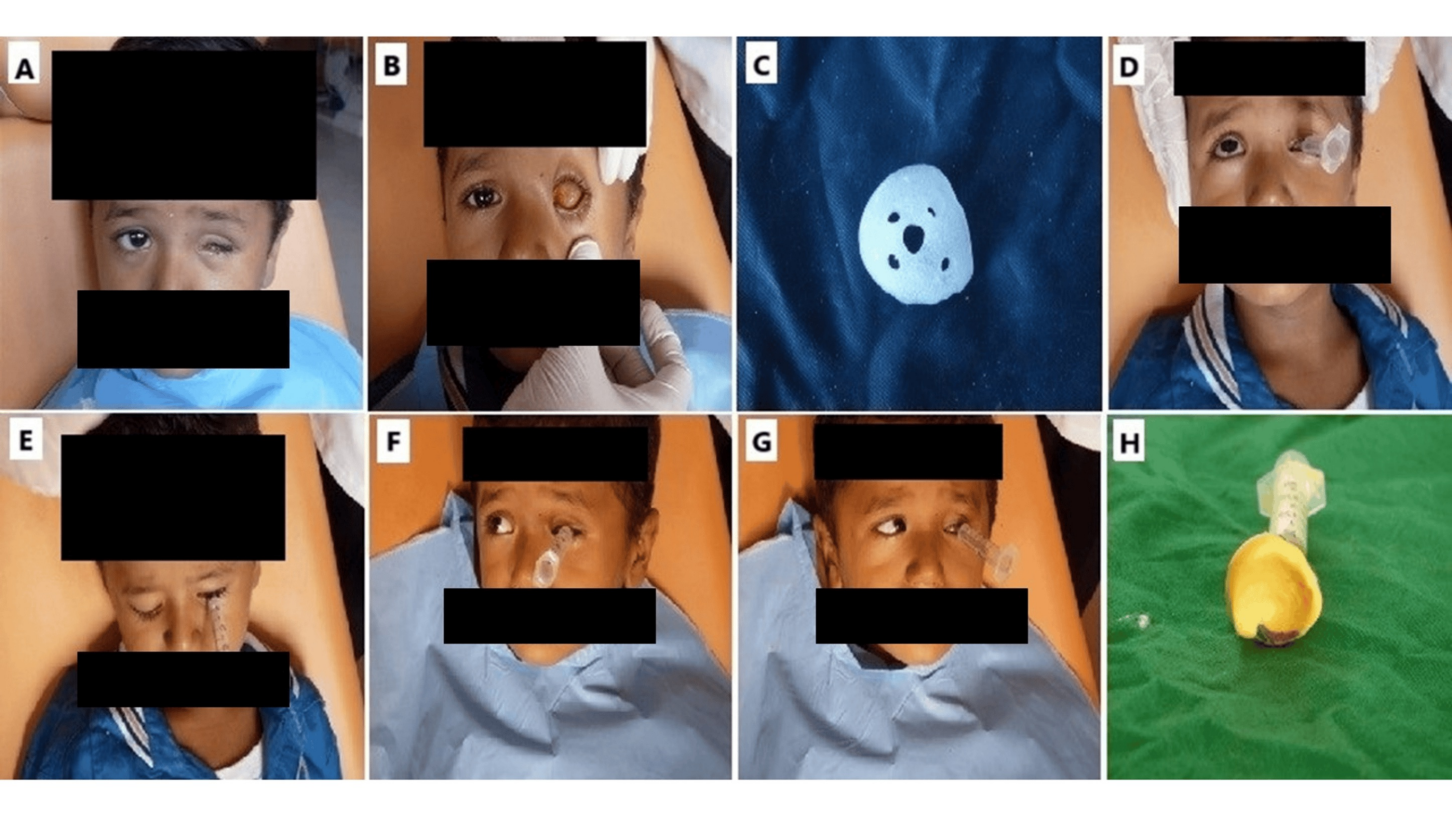
A. Pre-prosthetic photograph; B. Examination of socket bed; C. Custom tray fabrication; D. Upward functional movement during final impression; E. Downward functional movement during final impression; F. Right lateral functional movement during final impression; G. Left lateral functional movement during final impression; H. Final impression of the socket bed. It shows a pre-prosthetic photograph to document the initial condition before prosthesis fabrication. The socket bed is examined to assess its condition and inform the prosthetic planning. A custom tray is then fabricated, specifically designed to fit the unique anatomy of the socket for accurate impression-taking. During the final impression, various functional movements are captured, including upward, downward, right lateral, and left lateral movements, to ensure that the impression fully reflects the dynamic behavior of the socket bed. The final impression provides a comprehensive representation of the socket’s anatomy and functional aspects, serving as the foundation for designing a well-fitting prosthesis.

A five-year-old male child presented to the Department of Prosthodontics and Crown & Bridge for prosthesis fabrication following the enucleation of his left eye (Figure 1A). Upon thorough evaluation of the medical records, it was noted that the child had undergone enucleation concerning the left eye after being diagnosed with retinoblastoma. ​ Due to the natural expansion of the socket bed with age, an ocular prosthesis was deemed the most suitable treatment option, as it allows for necessary adjustments over time to accommodate growth. The examination of the socket site showed healthy tissue, and the socket exhibited adequate mobility, making it suitable for fabricating a custom ocular prosthesis (Figure 1B).​ The treatment plan was carefully outlined to the guardian, who provided written consent.

Initially, the socket for the missing eye was cleaned to remove any debris and secretions. Upon lubricating the area of interest, an impression of the socket was made using an irreversible hydrocolloid that had been prepared with cold water and applied in a thin consistency via a syringe. A conformer was produced and appropriately modified to serve as a custom impression tray (Figure 1C). The child was instructed to focus on a distant object during the final impression process. The final impression utilized light body elastomeric impression material while performing various functional movements (Figure 1D, 1E, 1F, 1G). Once it had been set, the impression was carefully removed and evaluated for precision (Figure 1H).

**Figure 2:**
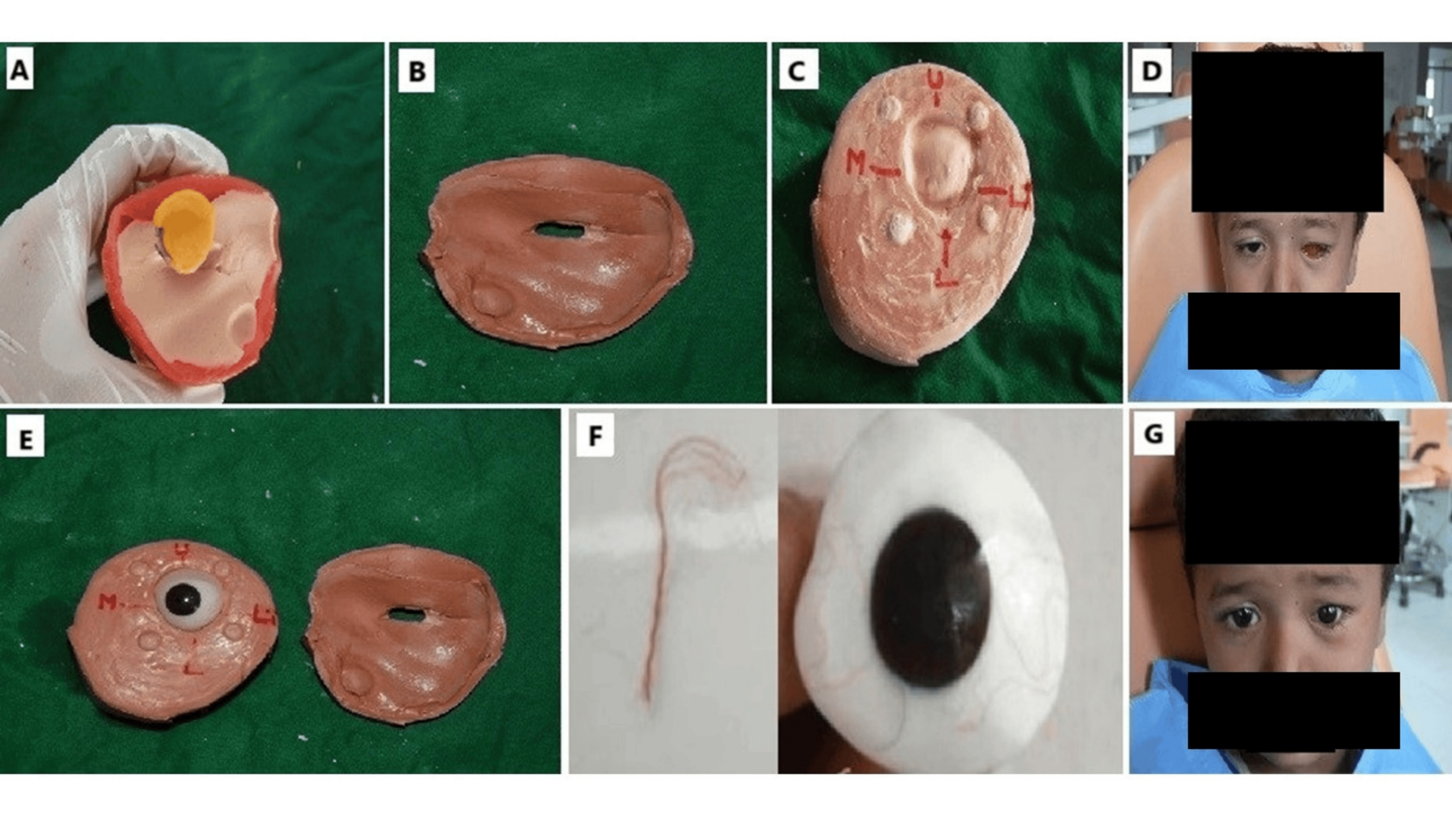
A. Final impression of socket bed with periorbital tissue.; B, C. Two-piece cast Mold; D. Wax pattern trial; E. Ocular prosthesis with incorporated iris button; F. Incorporation of red nylon fibres for providing natural appearance of sclera; G. Post-prosthetic photograph. The final impression of the socket bed is made, including the periorbital tissue, to accurately capture the detailed anatomy. This impression is used to create a two-piece cast mold, which serves as the foundation for fabricating the prosthesis. A wax pattern trial is then conducted to ensure proper fit and aesthetics before finalizing the design. The ocular prosthesis is crafted with an incorporated iris button, precisely positioned for a natural look. Red nylon fibers are added to mimic the natural appearance of the sclera, enhancing the realism of the prosthesis. The procedure concludes with a post-prosthetic photograph, showcasing the completed prosthesis in place.

Subsequently, a hydrocolloid impression was taken to capture the surrounding periorbital tissue (Figure 2A). This boxed impression was then poured into Type 4 dental stone to create a two-part mold (Figure 2B, 2C). After the cast had set, it was prepared for wax pattern fabrication. A separating medium was applied to the cast, followed by the pouring of modeling wax into the defective area of the cast. The wax pattern was sculpted to form an approximate contour of the eye. The fit of the wax pattern was verified in the child, particularly by evaluating the extension in the fornices (Figure 2D). The iris location was marked on the wax pattern while the patient focused on a distant object. The stock iris button was then incorporated into the wax pattern, and its centralization was verified using a graph. Adjustments were made to ensure the contour matched that of the remaining eye, focusing on fit and accuracy during both the fixed gaze and functional movements. The completed wax pattern was prepared for investment and processing. To ensure the iris remained properly oriented during the process, a stump made from clear self-cure polymethyl methacrylate (PMMA) was affixed to it. The wax pattern was then placed in a specialized ocular flask and dewaxed. The heat-cured tooth-colored PMMA in the appropriate shade was mixed and packed into the dewaxed mold cavity.

After packing, the flask was left to undergo bench curing before being placed in boiling water for an hour to complete the curing process.The final prosthetic pattern was retrieved from the flask and completed (Figure 2E). The surface of the prosthesis was uniformly reduced by approximately 0.5 mm. To add realism, red nylon fibers were incorporated to represent vessels on the scleral surface (Figure 2F) before reinvesting using heat-cured clear PMMA. Once the final prosthesis was extracted, it was finished and polished. The completed prosthesis was then disinfected and placed into the ocular socket for fit assessment (Figure 2G). The guardian was advised to moisten the prosthesis before insertion, with instructions that it could be worn both day and night, requiring daily cleaning with soap and water. The importance of routine follow-up appointments was also emphasized.

Case 2

Orbital Prosthesis

**Figure 3:**
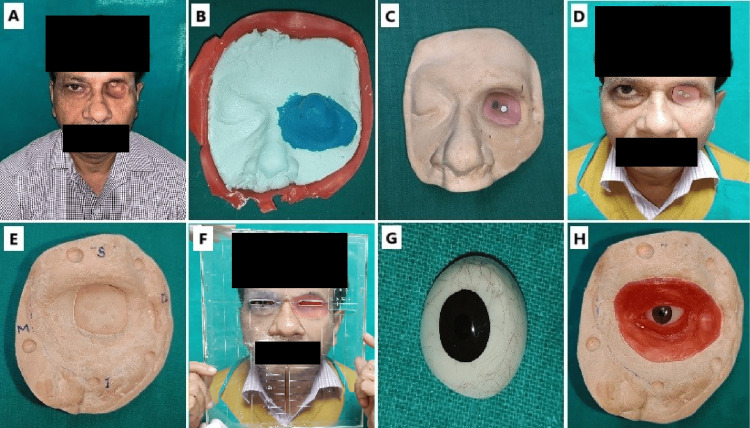
A. Pre-prosthetic photograph; B. Impression of the moulage; C. Fabrication of acrylic attachment; D. Try in of acrylic attachment; E. Working cast model; F. Iris location using trubyte tooth indicator; G. Fabrication of conformer; H. Wax carving. A pre-prosthetic photograph is captured to document the initial condition. An impression of the moulage is then made to capture the detailed anatomy, followed by the fabrication of an acrylic attachment that is tried in to ensure proper fit and alignment. A working cast model is created as a precise replica of the anatomical structures, which aids in further stages of prosthesis fabrication. The iris location is determined using a trubyte tooth indicator to achieve accurate positioning. A conformer is fabricated to maintain the shape of the socket, followed by detailed wax carving to refine the prosthesis design before finalization.

A 55-year-old male reported to the Department of Prosthodontics and Crown & Bridge for evaluation and treatment following the loss of his left eye. The patient’s history revealed that an orbital exenteration had been performed two years prior due to a low-grade myxofibrosarcoma. Upon examination, a significant orbital defect was observed on the left side (Figure 3A). Additionally, a pronounced bony undercut was noted superiorly, which could aid in the retention of the prosthesis. There was no history of pain, redness, suppuration, or discharge, and the site was completely healed. Initially, a three-dimensional reconstructive computed tomography (CT) scan of the head and neck was performed to plan for implant-supported prosthesis; however, the scan revealed insufficient bone in the targeted area for implant placement. Given these findings, a two-piece magnet-retained silicone orbital prosthesis was planned to enhance the patient’s social appearance and improve his quality of life. The area of interest was lubricated, and an impression of the defect was made using light-body polyvinyl siloxane impression material. The impression was then made for the remaining facial area captured using irreversible hydrocolloid (Figure 3B).

Wet gauze was spread over the hydrocolloid before it was set, and the entire impression was reinforced with quick-setting dental plaster. This impression was cast in Type IV dental stone to create a model. An acrylic component was fabricated to secure retention from the undercut area, and this acrylic piece formed part of a two-piece prosthesis (Figure 3C). To enhance the retention of the orbital prosthesis, two samarium-cobalt (SmCo) magnets were employed. The acrylic component was then tried in the patient (Figure 3D). A second impression of the defect was then made using light-body polyvinyl siloxane elastomeric material, with the acrylic component in place, to create the working cast (Figure 3E). A wax-up was done on this working model to form the base for the conformer. Chairside marking for the iris location was done on the patient using a trubyte tooth indicator, with all coordinates referenced to the opposite eye (Figure 3F). Following this, a conformer was fabricated using autopolymerizing resin, and the iris and pupil were colored (Figure 3G). The conformer was positioned within the wax pattern, and symmetry was reassessed using the trubyte indicator. Once the position was confirmed, the wax carving of the periorbital tissues was performed (Figure 3H).

**Figure 4:**
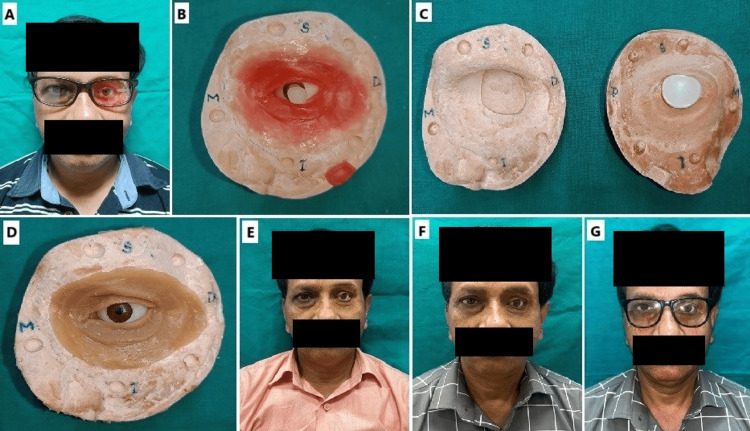
A. Try in of orbital prosthesis; B. Waxed up orbital prosthesis; C. Dewaxing of the mold; D. Cured silicone-based orbital prosthesis; E. Extrinsic staining of orbital prosthesis; F. Post-prosthetic photograph without spectacles; G. Post-prosthetic photograph with spectacles. The try-in of the orbital prosthesis is done to assess the fit and aesthetics. This is followed by waxing up the prosthesis to finalize its contours and details. The mold is then dewaxed to prepare for the curing process. A silicone-based orbital prosthesis is cured, creating a durable and lifelike final product. Extrinsic staining is applied to the prosthesis to enhance its natural appearance, matching the surrounding skin tones and textures. The process is documented with post-prosthetic photographs, both without and with spectacles, to showcase the prosthesis's integration and aesthetic outcome.

A try-in of the waxed-up prosthesis was conducted to verify symmetry (Figure 4A). The wax-up was completed on the cast, followed by attaching a stalk to the center of the acrylic conformer to hold it in place during dewaxing (Figure 4B). Dewaxing was then performed (Figure 4C), and the molds were thoroughly cleaned. A vent hole was created between the two parts of the cast to allow excess material to flow out and to facilitate separation. Room temperature vulcanized medical-grade silicone material was manipulated and intrinsically stained the chairside. Once a shade slightly lighter than the patient’s skin tone was achieved, the mixed silicone was packed into the mold. The silicone was allowed to vulcanize at room temperature for 24 hours, after which the casts were separated. This process yielded the cured silicone-based orbital prosthesis (Figure 4D).

The conformer was then separated from the stalk, and a try-in of the prosthesis was conducted on the patient. Extrinsic staining was performed chairside to achieve the desired shade (Figure 4E). Once the shade was finalized, a sealant was applied to lock in the pigmentation. After sealing, artificial hair was sewn to create eyelashes and part of the eyebrows. The final prosthesis was evaluated both with and without spectacles to check the margins (Figure 4F), and a light reflection test was conducted to verify the symmetrical positioning of the pupil and iris (Figure 4G). A follow-up was conducted after six months, during which the shade was reassessed. The patient was confident in rejoining social activities and satisfied with the aesthetic outcome.

## Discussion

For years, artificial eyes have been replaced with either stock or custom prostheses. However, a custom-made prosthesis is frequently essential, as it provides a more precise and aesthetically pleasing outcome, especially for patients who have undergone enucleation or exenteration. Practitioners can employ a variety of effective techniques in the rehabilitation of individuals requiring custom prostheses [6].

While implant-retained prostheses play a critical role in enhancing treatment outcomes, conventionally retained ocular or orbital prostheses continue to be practical, straightforward, cost-effective, and reliable alternatives [7]. Although these prostheses do not restore vision, they serve as suitable aesthetic replacements for patients, contributing significantly to psychosocial well-being and self-esteem [8]. In pediatric cases such as the one described in this report, it is vital to maintain the size of the ocular defect to prevent complications, such as microphthalmia, which may make future restoration increasingly challenging [9].

As a child matures, the ocular prosthesis will require replacement or modification to accommodate growth. Indications for updates include inappropriate prosthesis rotation within the socket, loose fit, corneal decentration, cosmetically significant ptosis, or discoloration of the prosthesis [10]. The size of the prosthesis is gradually enlarged to align with the child's growth over several years until the socket is fully developed, which typically occurs by the age of 12. During this growth phase, the soft tissues stretch, facilitating the development of the fornices for a more natural appearance [11]. Over the course of approximately 12 years, adjustments to the prosthesis should be made based on clinical observations and in coordination with the patient's facial growth to support the development of both the soft and hard tissues of the ocular socket.

​Room temperature vulcanized (RTV) medical-grade silicone is highly valued in orbital prosthesis fabrication for its excellent biocompatibility, flexibility, and ability to closely match skin tones, giving a lifelike appearance [12]. However, it degrades, discolors, and loses durability over time. Routine upkeep and timely replacements are recommended to address these limitations. Samarium-cobalt magnets for retention offer strong magnetic retention and stability but may suffer from corrosion and weakening [13]. Properly encapsulating the magnets in biocompatible materials like PMMA can minimize these issues, and routine checks of retention strength are vital for maintaining long-term performance.

The success rate for both orbital and ocular prostheses is generally high, reinforcing their value in ocular rehabilitation.​ Proper management and timely modifications can ensure optimal fit, comfort, and aesthetics, thereby enhancing the overall quality of life for patients with ocular or orbital deficits [14]. Although these prostheses function adequately within their limitations, the advent of CAD-CAM technology offers new and improved alternatives for rehabilitating such defects [15].

## Conclusions

Custom ocular and orbital prostheses play a crucial role in enhancing the aesthetic appearance and emotional well-being of patients who have experienced ocular loss.​ These prostheses are meticulously tailored to match the individual's unique facial characteristics, which helps restore a sense of normalcy and improve social interactions. The creation of these prostheses involves comprehensive assessments, personalized design, and ongoing adjustments to accommodate the evolving needs of patients. This process emphasizes the importance of interdisciplinary collaboration among healthcare providers, ensuring that both physiological and psychological aspects are addressed effectively. Moreover, continued advancements in techniques and materials utilized in prosthetic rehabilitation promise to further elevate patient satisfaction and quality of life, ultimately enabling individuals to reintegrate into their social environments with confidence and dignity.
